# Community pharmacists’ role in preventing opioid substitution therapy-related deaths: a qualitative investigation into current UK practice

**DOI:** 10.1007/s11096-019-00790-x

**Published:** 2019-02-15

**Authors:** Ramesh Yadav, Denise Taylor, Gordon Taylor, Jenny Scott

**Affiliations:** 1 Department of Pharmacy and Pharmacology, University of Bath, C/O: 5 West 3.26, Claverton Down, Bath, BA2 7AY UK; 2 Department for Health, University of Bath, Bath, UK

**Keywords:** Buprenorphine, Community pharmacy, Intoxication, Methadone, Opioid-related death, Opioid substitution therapy, United Kingdom

## Abstract

*Background* Opioid substitution therapy involves prescribing of medical substitutes like methadone and buprenorphine to patients who are addicted to opioids. The majority of opioid substitution therapy dispensing in the UK is done by community pharmacists and they often see the patients on daily basis. It is unknown to what extent community pharmacists implement the policy to prevent overdose in patients receiving such treatment. *Objective* To explore what UK community pharmacists think about their role in preventing opium substitution-related deaths, their understanding of the risks associated with this substitution therapy and their views on what else community pharmacists could do to reduce such deaths. *Setting* Twenty four community pharmacists from two areas in UK (Worcestershire and Bath and North East Somerset). *Method*  Between January and March 2013, community pharmacists providing opoin substitution therapy were interviewed in their pharmacy, using semi-structured interviews. Interpretative Phenomenology Analysis was used to analyse the data. *Main outcome measure* Thematically organised description of professional practice as reported by the participants against the clinical/practice guidance for opioid substitution therapy in UK. *Results* While participants felt their role to be essential in providing the service, they did not feel part of an integrated system. Participants’ ability to act in risk situations was affected by their knowledge, confidence in intervening in such situation, as well as the support they receive in providing the service. *Conclusion* Participants reported large differences in how ‘opioid substitution therapy’ services are provided in community pharmacy. Lack of knowledge among some pharmacists and lack of support in providing the service resulted in some patients at high risk not having their risks acted upon.

**Electronic supplementary material:**

The online version of this article (10.1007/s11096-019-00790-x) contains supplementary material, which is available to authorized users.

## Impacts on practice

- Pharmacists providing opioid substitution therapy service should receive mandatory standardised training, and mechanisms should be in place to monitor their training and competency; existing mechanism like the ‘Centre for Pharmacy Postgraduate Education CPPE self-declaration of competency’ can be adopted.
- Practice guidance to pharmacists should be clear and specific to deal with the challenges faced by community pharmacists in providing the service (for example identifying intoxication, dose withholding, out-of-hours referrals, dealing with security concerns).
- Information sharing between the treatment centres and the community pharmacies should be promt and standardised to ensure clarity and consistency.

## Introduction

Opioid substitution therapy (OST) is the mainstay of pharmacological therapy for opioid dependence in the United Kingdom (UK) [1]. The majority of this treatment is provided in the community setting with prescribing being undertaken by either community drug teams or general practitioners. Methadone and buprenorphine are the most commonly prescribed OST treatments [2]. Community pharmacists (CPs) are increasingly involved in providing services related to OST, including dispensing, supervising consumption and giving advice on preventing overdose [1, 3, 4]. Dispensing of OST can be undertaken at a registered pharmacy, but supervised consumption and needle and syringe provision, two other key harm reduction intervention, require a service level agreement (SLA) with local commissioners. While commissioners may require evidence of training in this area, no formal qualification is required.

Opioid-related deaths have received significant attention in both the literature and media in recent years. In 2016 they accounted for over half of the drug related deaths in England and Wales. Mostly these deaths are the result of accidental poisoning and involve multiple drugs (mostly heroin and/or morphine) and alcohol [5]. Methadone was listed as one of the drugs in 691 deaths in UK in 2015 as compared to 565 deaths in 2010 [5–7].

Strang et al. [3] demonstrated that a shift in methadone treatment practice, introduced in the mid-1990s, has prevented methadone-related deaths increasing as prescribing has increased. In brief, this policy involves supervising the consumption of OST doses (which only became common practice in later years [8]), withholding OST dosing of intoxicated patients e.g. with alcohol, re-titrating patients who have missed more than 3 days’ OST treatment, and providing overdose prevention advice on completion of detoxification [1]. Most OST treatment in the UK is provided in the community, and the majority of CPs undertake OST dispensing [8, 9]. As they often see patients on a daily basis, they are the main healthcare professionals responsible for ensuring that OST is undertaken in accordance with the guidelines of preventing overdoses i.e. withholding dose from intoxicated patients and those who have missed three or more doses and referring them to the prescriber and giving advice on preventing overdose [1]. According to a study, between 2008 and 2011, the English public treatment system for opioid use disorder prevented an average of 880 deaths each year from opioid-related poisoning [10]. While continuing in OST is associated with reduced risk of death [11], it is unknown whether pharmacists could do any more to reduce OST-related deaths.

A 2015 report by the Advisory Council on the Misuse of Drugs (ACMD) [12] raised concerns about the variable quality of drug treatment services at a local level in England. It identified variations in the quality and skills of staff and the culture of the local treatment system. A 2016 report by Public Health England (PHE) 2016 [13] recommended actions to reduce drug-related deaths, of which opioid-related deaths are a significant component. Among several other measures, it recommended the strengthening of clinical governance and workforce competence to reduce drug related-deaths. While these and other reports suggest changes to the local treatment systems in general, they remain silent on the community pharmacy aspects of the service. There has been very little work done to understand the role of community pharmacy in minimising opioid-related deaths. Based on the limited literature published and anecdotal evidence, it is hypothesised that the policy to prevent OST-related deaths is not being fully implemented by pharmacists, and that more could be done to reduce these deaths.

## Aim of the study

To explore what UK community pharmacists think about their role in preventing OST-related deaths, their understanding of the risks associated with OST and their views on what more community pharmacists could do to reduce such deaths.

## Ethics approval

Ethical approval was obtained from the Research Ethics Approval Committee for Health (REACH), University of Bath (REACH reference number: EP 12/13 18). All participants were required to give signed consent before the interviews were conducted.

## Methods

Qualitative methods were used primarily because of the lack of any significant research in this area of pharmacy service. Semi-structured, face to face interviews were considered appropriate for the study as they allowed for the collection of enriched data on personal experiences, understanding and views. Due to their sensitivity, these experiences and views might be difficult to express during other data collection methods like focus groups. CPs were recruited from two different sites: Bath and North East Somerset (BANES) and Worcestershire. These two locations provided a good mix of rural and urban pharmacies. All community pharmacies (n = 138) in these two study sites were sent an invitation pack inviting them to take part. The invitation pack was addressed to ‘The Responsible Pharmacist’. An expression of interest form included seven demographic questions to allow for purposive sampling. These markers were adapted from the analysis of GPhC pharmacist register 2011 [14]. From the responses received (n = 31) participants were selected using maximal variation sampling to gain a broad sample based on age, gender, years of experience and role within the pharmacy. All had experience with substance misuse service provision. The final number of participants was guided by the principle of data saturation, which was achieved at 24 interviews. No financial incentive was offered for participating in the research.

An interview guide was first drafted based on an initial literature search and was reviewed following two pilot interviews. The interviews were digitally audio-recorded and transcribed verbatim. Each transcript was de-identified, anonymised and checked for typographical errors and any missing information.

RY undertook the interviews. All transcripts were coded by RY and a sample by DT to confirm the coding process, using interpretative phenomenology analysis (IPA). IPA allows researchers’ interpretation of the experiences lived by the participants. RY is a part time community pharmacist, which was made known to participants. IPA was chosen as it allowed the participant to relate to the researcher in terms of their shared professional roles, allowing professional nuances and language to have a shared understanding. It also allowed the experience of the researcher to be used in the interpretation of data to understand the lived experience of participants [15]. The analytical process started with listening, reading and rereading of the transcripts. The transcripts were then coded to specific words or phrases that reflected the lived experience of the participants. The emergent codes were discussed among research team members (RY, DAT and JS) to reaffirm rigour and consistency, allowing convergence and divergence to be identified. A pattern started to emerge as the coding progressed. When a new code emerged, previously coded transcripts were reviewed to check if such views were expressed by other participants. The process was one of constant iteration to ensure consistency in coding. The codes were then organised into themes and subthemes representing a particular participant experience of OST provision. QSR NVivo 10 software was used as a data management tool. For the purposes of reporting, participant pharmacists have been assigned a pseudonym, with actual job role, gender and years of practice experience stated.

## Results

Participants ranged from newly qualified (less than 1 year) to those with experience of greater than 30 years. Participants all provided OST service, but had varied amounts of experience of this service. They had varied roles within the pharmacy. There were 14 female and 10 male participants. While most participants were UK graduates, two were from Europe and a further two from India. All graduates are required to register with General Pharmaceutical Council (GPhC) before they can practise in the UK.

The interviews were conducted in the consultation room of the participants’ pharmacy and lasted between 35 to 60 min.

Three key overarching themes emerged from the analysis in relation to the aims of the study:

1. Organisational challenges of providing OST service.
2. Managing risk in practice.
3. Behavioural and environmental impact on patient care.

### Organisational challenges in providing OST service

Though participants, in general, felt their role was essential in providing OST services within the existing model, most did not feel part of a co-ordinated system. While they realised the importance of their frequent interaction with the patients (almost daily to once a week) there were also concerns and frustration that the potential of such high frequency interaction were not capitalised upon because of this lack of coordinated working. These views of detachment were underpinned by personal experiences of inability to contribute to clinical decision making; difficulty in prompt communication and lack of support in providing OST. This is illustrated in the quote below by Jill when she describes the lack of communication prior to receiving a prescription.

We do try as much as I could to get involved in the patient care but I am not given much scope, so we only get told what dose to give, when and where, sometimes we even are not updated about the dose change until we get the script.

Jill, Pharmacy manager, F, 1.5 yrs experience

Sharing of information between treatment teams (prescribers) and pharmacies can be crucial in clinical decision making; thus delay in communication and problem solving caused frustration for many participants. They also expressed concerns about the support available to them when making clinical decisions (for example dose withholding from an intoxicated patient), particularly during out of hours.

I think we have a much prompter response if it is a green [non-OST general practice] prescription from a doctor, we would get a call within 15 min or the next half an hour… so I don’t understand why we can’t get to speak to a professional about the blue one [blue refers to two weekly OST prescriptions] and very unlikely that we can get a doctor on the phone, very, very unlikely it takes ages to get a doctor on the phone, you get a worker [support worker] all the time in the [drug] clinic.

Josephine, Locum, F, 8 yrs experience

Participants expressed views about variation in the service delivered to OST patients, some were concerned about non-equitable services to OST patients, as expressed in the example below.

There are different ways of looking at it but I don’t think it’s a standardised service for everyone, I think it’s hit and miss as to which pharmacy you go into, which pharmacist is on duty you know so yeah I think it could do with some sort of work across the board really.

Amanda, Second pharmacist, F, 24 yrs experience

One aspect of the variations in practice experienced by the participants was in the way the OST service was commissioned and organised in primary care. Different service providers have different working practices (for example the requirement for community pharmacists to report back on any missed dose by patients) thus leading to regional variations. For Lee, the commissioning of the service through different providers meant different expected outcomes leading to provider variations.

I suspect that different providers would want maybe different things, but if there was a sort of a national framework that they understood this was what was happening…

Lee, Pharmacy manager, M, 20 + yrs experience

### Managing risk in practice

Participants expressed a varying degree of awareness about the prescription-related factors that have potential to harm or kill a patient receiving OST.

…clients who are showing signs of withdrawal possibly needs referring back because they are not getting adequate treatment and the risk of them using additional substance is going to be high.

Amy, Pharmacy manager, F, 1.5 yrs experience

While most participants identified the innate overdose danger associated with OST, others showed little recognition of risk factors. Where participants recognised potential risk situations they did not necessarily act to mitigate the risk. Such actions or inactions were underpinned by lack of confidence and knowledge in dealing with the issues, misinformation, training gaps and no awareness of clinical guidance.

Some participants reported that they had not taken any remedial action in cases where there were threats to the wellbeing of the patient, for example; intoxication of patients receiving their OST treatment. Generally, this non-intervention was explained by participants perceiving that the patient would not be harmed as they were used to risky drug taking habits.

To me the danger is, ‘is this dose going to cause him harm if he takes it?’ and I have always thought well that’s pretty unlikely. I think if he can manage to get here and he can get down the stairs (pharmacy located downstairs) and he isn’t falling over then he can probably take this without harming himself, whether his treatment is of any benefit to him is another issue obviously.

Joseph, Pharmacy manager, M, 32 yrs experience

Joseph, as many others, also reflected on the complexity faced by pharmacists in ascertaining the clinical appropriateness of OST prescriptions and suggested how lack of professional confidence could lead to inaction by pharmacists in risky situations. Largely, participants acknowledged that pharmacists failed to undertake a clinical assessment of OST prescriptions.

…we’re supplying them with daily or almost daily medication. We theoretically have a duty to question the prescribing or the treatment if we thought it was in effect more dangerous or counter-productive or whatever, but that requires us to have the confidence to suggest something to the people we perceive to be experts in this field and that’s quite difficult.

Joseph, Pharmacy manager, M, 32 yrs experience

The issues of professional confidence were reflected equally among newly qualified and those with several years of pharmacy experience. While some were more forthcoming in accepting their professional shortcomings others were hesitant and reverted to using plural nouns (‘we’ rather and ‘I’) to reflect a collective approach in adopting such practices. The switching between singular and plural noun is evident in the two statements above.

Amanda in her quote below, also uses ‘We’ to reflect a collective responsibility in not performing clinical checks on OST prescriptions which she puts down to lack of information.

We can look at them (OST prescription forms) and think gosh this is high (dose) but then we don’t know their drug history, we are not told of their drug history, often we can’t do clinical checks because they might come and collect their methadone from us but they might get their other prescriptions from another pharmacy and they’re not always willing to tell us what they are taking so in that respect no there’s not (clinical check) but then unless we have access to their clinical records I don’t see that we can do much more.

Amanda, Second pharmacist, F, 24 yrs experience

Participants often shared their experience of OST in comparison to non-OST prescriptions, thus giving an insight into differences in professional thinking that happens while caring for OST and non-OST patients. Despite giving more time and attention to process an OST prescription, pharmacists omitted the critical step of clinical checking, as legal and dispensing accuracy was their priority. The views expressed by the two participants below encapsulated this attitude among participants.

To be honest, I mean this is really being honest. I know what the correct answer is; that you would check against what they take and then you make sure that it is clinically appropriate and you check that the dose is not overdose that kind of thing, I don’t think that happens in practice…

Rachel, Relief Pharmacist, F, 3.5 yrs experience

I think pharmacists unfortunately, tend to think more of the accuracy. I don’t think you probably have sometimes too much time to go further than that and probably it is quite sad but it is how it is…. You are making me reflect on my practice which is quite important and interesting.

Jasmine, Pharmacy manager, F, 12 yrs experience

The perceived lack of training and guidance on OST were referred to by many of the participants. While most of the participants seemed unaware of the existence of clinical guidance relevant to OST, those who knew of the guidelines expressed concern over the lack of clarity within existing guidance. Training and guidance on dealing with more challenging aspects of OST in community pharmacy, such as dealing with intoxicated patients, missed doses and dose withholding, were identified by participants as necessary to improve their role in minimising OST-related deaths. Peter, who had been qualified for 2 years, found the guidance to be ‘a grey area’ when deciding on dispensing to patients under the influence and made decisions based on personal experience rather than on any guidelines.

I don’t think we have been provided [with] enough training with how do deal with if an addict was under the influence, it is a bit of a grey area really. You are not told what the protocol is. You don’t really know, I mean it’s, you are using your sort of clinical judgement really your benefits against the risks aren’t you? You are just going to decide for yourself whether or not to supply it.

Peter, Second Pharmacist, M, 2 yrs experience

### Behavioural and environmental impact

Participants related their decision making around OST with the work environment they operated in. Many participants shared examples of specific situations where, at the least, their approach towards an OST patient would be different, and more critically, their clinical decision making might be compromised when compared to a non-OST patient, in the management of risk. Workload was cited as a factor that could compromise participants’ clinical input in the care of OST patient. Some participants gave examples of situations when they might provide diminished care with potential to harm the patient.

To be honest the workload so if I’m very very busy I’ll be more inclined to just give it (dispense to an intoxicated patient) and give them a word of caution ah for me it’s probably workload how many things I’ve got to do at that moment in time, if I’m very stressed that’s what I would do… I just think there’s so many pressures that pharmacists come across and I think this probably does happen in general. I don’t think I’d be alone in admitting it.

Rita, Pharmacy manager, F, 4.5 yrs experience

The statement above confers to the idea of a collective approach, as referred to under risk management, among the participants on otherwise professional shortcomings.

Stigma or stigmatised behaviour by participants, patients and others involved in OST services were evident in many of the participants’ narratives. Most participants acknowledged the stigma associated with this aspect of healthcare and sought to avoid stigmatised behaviour in their own practice. However, a minority of the participants admitted how their individual negative preconceptions about OST affected their service provision.

I know some people see a methadone patient as a different sector of patients, that they’re ones that you try to get out of the shop as fast as possible, they’re ones you don’t want to have any interaction with.

Tracey, Pharmacy manager, F, less than 1 yr experience

In the quote below, Joseph is keen to point out the importance of treating his patients equally; nevertheless, he is also aware how his underlying beliefs might not necessarily lead him to do so.

I think it’s extremely important for me to treat everybody with the same respect and in a way that’s not in any way prejudicial, not always that easy and we do have hidden underlying prejudices and there are all kinds of things that we can often not avoid which make us behave in a different way to one person than to another.

Joseph, Pharmacy manager, M, 32 yrs experience

The unavoidability of the effect of preconceptions about people who use drugs was expressed more bluntly by other participant, Matt, when he said; ‘It is a stereotype, but a stereotype is a stereotype for a reason.’

The comfort of familiarity among pharmacist, patients and others involved in OST was referred to by the participants as one of the reasons for some of the deaths related to OST. Frequent exposure in dealing with opioid substitutes can lead to a false sense of security and diminished responsiveness in identifying and acting on risky situations. Such actions put patients and others who might gain access to the drugs at increased risk and was highlighted, for example in relation to cases where children have been the victim.

I think part of the problem is maybe it is because when you have been on methadone so long you fail to see it a potentially dangerous drug because it is something you have every day and you sort of get familiarity for its content sort of thing and it is like ‘it is alright for us it will be alright for the baby

Chris, Owner, M, 23 yrs experience

While participants reflected positively on the good relationship they develop with their OST patients over time, there were also concerns of security of the pharmacy and its staff. Participants shared personal experiences and examples where such security concerns affected their professional decision making thus compromising patient safety. While some female participants shared how their gender and personal security concerns affected professional decision making, such concern, however, were not limited to female participants. This is illustrated by Susan, who describes how her decision to withhold an OST dose from an intoxicated patient may be influenced by her perceived risk of aggression and her perception of her own vulnerability.

It is one of the hardest to be honest (dose withholding in intoxicated patients) because quite often umm those who are the heavy drinkers are also quite often the most aggressive so being a young small female the last thing I particularly want is to put myself at risk (by withholding the dose) umm so it is a case by case scenario

Susan, Pharmacy manager, F, 5.5 yrs experience

## Discussion

This study demonstrates that while CPs can identify the innate overdose danger associated with OST there are variations in their understanding of risk and their willingness and ability to take remedial actions where concerns exist. It also demonstrates the variability in the provision of services provided by community pharmacists to OST patients. More importantly, it highlights how some patients might be at greater risk of OST-related deaths due to the action (supplying dose to an intoxicated patient) or inaction (missing clinical check of OST prescriptions) of CPs.

While critical to the delivery of OST services in community based care, participants considered that they are seen as the supplier of the medication and not part of the integrated treatment team. Input by pharmacists in OST services seems dependent on the interest and proactive initiative of individual pharmacists. While successive reports and guidance [16, 17] have recommended better integration of CPs in the treatment service to minimise OST-related deaths, the experience of the pharmacists participating in this research suggests that in their localities, this has not happened.

In the absence of standardised communication and feedback mechanisms between the treatment teams and CPs, current information sharing is patchy, with different working practices emerging locally. The ACMD report 2015 [12] highlighted the variability of the service due to different providers. While this report does not comment on the provision of OST services in community pharmacies, the findings of this research nevertheless, are in line with that of ACMD findings.

In the absence of mandatory professional training, and the variable extent to which OST is taught at undergraduate level in UK universities [18] a situation exists where the service is provided by pharmacists with little or no exposure and understanding of the service. Some pharmacists did not use the same in-depth clinical checking to establish prescription safety as is completed for non-OST dispensing (for example, not checking patient medication record for possible interactions, not talking to the patient about side effects and outcomes). CPs’ decision to dispense to intoxicated patients has also been reported by other researchers [9]. CPs’ professional decision-making was influenced by workload, safety concerns and the stigma attached to OST. The negative effect of aggressive or inappropriate patient behaviour on pharmacists’ decision-making has also been reported by other researchers [19, 20]. This is one area where practice urgently needs appropriate guidance and education to enable change.

Part of the aim of this study was to identify what more could be done to reduce OST-related deaths. Familiarity about the risks associated with OST and a misguided comfort of other pharmacists adopting a similarly compromised practice, prevented participants from intervening in risky situations. Hesitations, use of pronoun ‘We’ and reference to experiences of some other pharmacists in expressing difficult situations were noted among some participants in distancing themselves from challenging situations. Lack of training and confidence in dealing with OST-related issues, misinformation, training gaps and lack of guidance were identified by the participants as reasons for not intervening in situations where a patient could be at risk of harm. Recent developments in practice include the provision of take home naloxone by drug treatment services and pharmacies to people who use drugs and their families and carers to treat overdose. This study implies that the opportunities for proactive take home naloxone supply may not be fully utilised until issues of confidence and training are addressed. More work is needed to establish if this is indeed the case around naloxone supply.

### Implications for policy

While drug policies have evolved to reflect the political will of the incumbent government [21, 22] the involvement of CPs in OST has also greatly increased in recent years [9]. Policy makers should be aware of the challenges faced by community pharmacists in providing OST services at the frontline of service provision. Drug policy should become more inclusive to harness the strategic position of CP in monitoring and supporting patients to minimise OST-related deaths. Local commissioning of the OST service and the involvement of different service providers makes it difficult to have consistency at local and national level. A ‘National Commissioning Framework for OST Services’ should be adopted to streamline service delivery across all localities. As stated in the introduction, while local commissioners may require evidence of continued professional development, there is no national certification of pharmacist who are suitably trained to provide this service. National certification mechanism currently exists for other pharmacy services like flu vaccination and it would seem prudent that this gap in OST service provision is addressed.

### Limitations and suggestions for further research

This is the first published qualitative research to describe community pharmacists’ opinion on OST-related deaths. While the data generated are rich in content it only involved participants from two localities thereby only reflecting the OST practice in these areas. A national study, a quantitative survey, to test if the issues identified in this research are more widespread would add significantly towards optimising the role of the CP in preventing OST-related death.

## Conclusion

Participants reported large differences in how OST services are provided in community pharmacy. Local commissioning of the service and the involvement of different service providers makes it difficult to have consistency at local and national level. A lack of standardised service protocol, training requirements and differences in policies from different commissioners and service providers exaggerated these variations. Lack of knowledge among some pharmacists and lack of clear guidance and support in providing the service resulted in some patients at high risk of OST-related deaths not having their risks acted upon. Thus, a national standard of mandatory training for pharmacist, harmonisations of local policies, better integration of CPs in the service provision and support to CPs providing the service could improve the role of community pharmacists in preventing OST-related deaths.

## Electronic supplementary material

Below is the link to the electronic supplementary material.

Supplementary material 1 (DOCX 15 kb)
