# Supplementary material for: Community pharmacists’ role in preventing opioid substitution therapy-related deaths: a qualitative investigation into current UK practice
Source: Int J Clin Pharm. 2019 Feb 15;41(2):470–7. doi: 10.1007/s11096-019-00790-x (PMC6509091; doi:10.1007/s11096-019-00790-x)
Supplement: Supplementary file 1 — Supplementary material 1 (DOCX 15 kb) [file 11096_2019_790_MOESM1_ESM.docx]

| **Participants** | **Age group** | **Employment status** | **Experience (Years)** | **Gender** | **Number of clients** | **Pharmacy location** | **Area** |
| --- | --- | --- | --- | --- | --- | --- | --- |
| **Amanda** | 40-49 | Second pharmacist | 24 | Female | More than 25 | Suburban | Worcestershire |
| **Amy** | 29 and under | Pharmacy manager | 1.5 | Female | 6-15 | Suburban | Worcestershire |
| **Ben** | 50-59 | Owner | 34 | Male | 5 or less | Urban | Worcestershire |
| **Carlos** | 29 and under | Pharmacy manager | 3 | Male | 6-15 | Urban | BANES |
| **Chris** | 40-49 | Owner | 23 | Male | 6-15 | Urban | BANES |
| **Edwin** | 29 and under | Pharmacy manager | 5 | Male | More than 25 | Urban | BANES |
| **Jasmine** | 30-39 | Pharmacy manager | 12 | Female | 5 or less | Rural | Worcestershire |
| **Jill** | 29 and under | Pharmacy manager | 1.5 | Female | 6-15 | Suburban | Worcestershire |
| **John** | 50-59 | Pharmacy manager | 30 | Male | 6-15 | Suburban | Worcestershire |
| **Joseph** | 50-59 | Pharmacy manager | 32 | Male | 5 or less | Urban | BANES |
| **Josephine** | 30-39 | Locum | 8 | Female | 6-15 | Urban | Worcestershire |
| **Lacey** | 40-49 | Second pharmacist | 18 | Female | 6-15 | Suburban | Worcestershire |
| **Laura** | 50-59 | Pharmacy manager | 30< | Female | 6-15 | Rural | BANES |
| **Lee** | 40-49 | Pharmacy manager | 20< | Male | 16-25 | Suburban | Worcestershire |
| **Linda** | 40-49 | Second pharmacist | 18 | Female | 6-15 | Suburban | Worcestershire |
| **Matt** | 30-39 | Pharmacy manager | 12 | Male | 6-15 | Suburban | Worcestershire |
| **Michelle** | 40-49 | Pharmacy manager | 18 | Female | 5 or less | Suburban | BANES |
| **Peter** | 29 and under | Second pharmacist | 2 | Male | 6-15 | Suburban | Worcestershire |
| **Rachel** | 30-39 | Relief pharmacist | 3.5 | Female | 6-15 | Urban | Worcestershire |
| **Rita** | 29 and under | Pharmacy manager | 4.5 | Female | 5 or less | Urban | Worcestershire |
| **Roger** | 30-39 | Pharmacy manager | 6 | Male | 5 or less | Suburban | Worcestershire |
| **Shirley** | 40-49 | Superintendent | 25 | Female | 5 or less | Suburban | Worcestershire |
| **Susan** | 29 and under | Pharmacy manager | 5.5 | Female | 6-15 | Rural | Worcestershire |
| **Tracey** | 29 and under | Pharmacy manager | > 1 | Female | 5 or less | Suburban | BANES |
